# Biomass-Based Sorbent with Superoleophilic from Ulva Prolifera for Oil Spill Cleanup

**DOI:** 10.3390/ma17225489

**Published:** 2024-11-10

**Authors:** Xiaotian Lei, Qiumin Kong, Yuqi Wang, Boping Yang, Dan Ouyang

**Affiliations:** 1 College of Textiles & Clothing, Qingdao University, Qingdao 266071, China; 2 Shiyan Key Laboratory of Quantum Information and Precision Optics, School of Mathematics, Physics and Optoelectronics Engineering, Hubei University of Automotive Technology, Shiyan 442002, China

**Keywords:** biomass materials, oil sorption, superoleophilic, Enteromorpho prolifera, surface modification

## Abstract

In this study, we demonstrate a new all bio-based adsorbent material by treating Enteromorpho prolifera (EP) fibers with tannic acid-ferric chloride complex and then grafting hydrophobic group octadecylamine. All raw materials are easily available, low-cost, and safe. The modified EP fibers have approximately 63.4 g $g^{-1}$ of oil absorption and 1.4 g $g^{-1}$ of water absorption, which is an 62.8% increase in oil absorption and an 82% increase in hydrophobicity over that of untreated EP fibers, respectively, exhibiting high hydrophobicity and oleophilicity. The affinity discrimination to water and oil enables hydrophobic algae candidate materials to separate oils and water efficiently, both in an oil–water mixture and a water-in-oil emulsion. In summary, the as-synthesized modified EP demonstrates a broad application prospect in the treatment of oil spill accidents and oily wastewater.

## 1. Introduction

Oil pollution produced by accidents in offshore oil fields, oil spills from tankers, and the discharge of oily wastewater is increasing, which brings serious threats to the marine ecological environment and human health. Studying effective methods for the treatment and recycling of oil spills is of great significance [1,2]. There are various methods for treating oil spills, such as physical, chemical, and biological methods [3,4,5,6]. Among these, the use of adsorbent materials, especially natural materials, has gained widespread attention for the removal and recycling of oil from sea surfaces and coastlines [7,8,9]. Natural adsorbents, such as plant fibers, algae, straw, or other organic materials, feature the advantages of abundant, cost-effective, easy to collect, environmentally friendly, and renewable, making them a promising solution for removing and recycling oil spills [1,9,10,11]. For instance, by using wood as a raw material, Wang et al. successfully constructed a superhydrophobic wood aerogel with a high oil uptake capacity of 20 g $g^{-1}$ and a remarkable separation efficiency of 99.5% [11].

Enteromorpha prolifera (EP) is a natural green algae species with very strong reproductive capacity. Since 2008, different sea areas, such as Qingdao, have experienced annual outbreaks of this algae, which pose serious aquatic ecological, environmental, and economic consequences. Therefore, it is urgent for finding the practical application of EP to mitigate its adverse effects. Currently, many researchers have explored Enteromorpha prolifera-based products with high added value, such as fertilizers, biofuels, food, and other applications [12,13]. These studies focus on utilizing the abundant biomass of EP to develop sustainable, eco-friendly products. Additionally, biomass EP fibers feature a hollow fibrous morphology and a multi-layer cellulose skeleton cell structure, making it highly effective for adsorbing various pollutants, including heavy metals, dyes, and oil, from water. The hollow and fibrous nature of EP provides a large surface area for adsorption, enhancing its capability to capture and retain contaminants. Additionally, the multi-layered cellulose skeleton contributes to its mechanical strength and stability, making it a suitable candidate for environmental remediation applications. By leveraging these properties, Enteromorpha prolifera can serve as an eco-friendly and sustainable option for water purification and pollutant removal, particularly in the context of oil spill treatment. Zhao et al. were the first to propose the use of fibrous algae as an oil absorber, modifying EP with dodecyltrichlorosilane to enable hydrophobic and superoleophilic properties [14]. More recently, Xue et al. prepared hydrophobic and superoleophilic EP by grafting n-octyltriethoxysilane, achieving maximum adsorption capacities of 11.4 g $g^{-1}$ [15]. Shi et al. developed a biological $C_{1}4H_{32}O_{3}$Si-modified Enteromorpha adsorbent using petroleum-degrading bacteria as a modifier, which is effective for removing diesel oil pollutants [16]. Boleydei et al. compared the absorption efficiency of crude oil and spent engine oil using algal biomass under various conditions, finding that the oil absorption by EP from seawater was more efficient than from freshwater [17]. Dong et al. prepared a superhydrophobic algae-based sponge by combining the growth of silicone nanofilaments, the deposition of silver nanoparticles, and the chemical vapor deposition of trimethylchlorosilane and tetrachlorosilane. These sponges exhibited high sorption capacities ranging from 11.7 to 17.1 g $g^{-1}$, fast sorption speeds (1.3–7.0 s to saturated), and excellent stability (including resistance to light, heat, chemicals, and mechanical stress) [18]. Ji et al. demonstrated an effective strategy for oil–water separation and water pollution remediation by incorporating Enteromorpha into graphene aerogel [19]. Dan et al. produced a hydrophobic Enteromorpha-derived carbon aerogel decorated with $NH_{4}H_{2}PO_{4}$, showcasing low density, rich porosity, and strong lipophilicity [20]. Wang et al. combined MXene, gelatin, and Enteromorpha to produce a biomass aerogel with excellent absorption capabilities for high-viscosity oil spills [10]. However, the aforementioned hydrophobic modification methods often involve toxic, harmful, and expensive organic compounds (e.g., polysiloxanes, organosilanes, MXene), or require significant energy inputs (e.g., chemical vapor deposition, high-temperature carbonization treatments). Therefore, there is a critical need for the development of economical, eco-friendly, and scalable approaches for fabricating biomass-derived sorbents with hydrophobic and oleophilic surfaces.

Tannic acid (TA), a natural polyphenol found in various plants and fruits, is characterized by its low cost, high reactivity, easy availability, good biocompatibility, and biodegradability. Due to its abundant hydroxyl groups, TA can effectively react with a wide range of substances, including alkaloids, enzymes, and metal ions, exhibiting a fast reaction rate. A key feature of TA is its ability to coordinate with iron ions ($Fe^{3+}$) in aqueous solutions, completing the reaction within just a few minutes at room temperature [21]. This process does not require complex equipment, making it accessible for various applications. Additionally, the free catechol groups in the TA-$FeCl_{3}$ complex can further react with amino or thiol molecules, allowing for additional functionalization and expanding its potential applications in various fields, including environmental remediation, drug delivery, and materials science [22].

Herein, we demonstrate the development of a novel all-bio-based adsorbent material by treating Enteromorpha fibers with a tannic acid-ferric chloride (TA-$FeCl_{3}$) solution and subsequently grafting hydrophobic octadecylamine (ODA) groups. The reaction between TA and hexahydrate $FeCl_{3}$ under alkaline conditions forms a cross-linked network structure that coats the surface of the EP fibers. The residual catechol groups in the TA-$FeCl_{3}$ complex can then undergo addition reactions with the amino groups in ODA, effectively grafting hydrophobic groups onto the EP fibers. All raw materials used in this process are readily available, low-cost, and safe. Notably, the modification alters the wettability of the EP fibers from superhydrophilicity to hydrophobicity, with added oleophilicity. The modified EP fibers demonstrate an oil absorption capacity of approximately 63.4 g $g^{-1}$ and a water absorption of 1.4 g $g^{-1}$, representing an 62.8 % increase in oil absorption and an 82% increase in hydrophobicity compared to untreated EP fibers. These hydrophobic algal materials exhibit excellent separation efficiency for oils and water, functioning effectively in both oil–water mixtures and water-in-oil emulsions. In summary, the synthesized modified EP fibers show significant promise for applications in the treatment of oil spill incidents and oily wastewater, offering an effective and sustainable solution to address environmental pollution challenges.

## 2. Experimental Section

### 2.1. Materials

All chemicals used were of analytical reagent grade, and were used as received, unless otherwise noted. Enteromorpha prolifera (referred to as EP hereafter) was collected from the coastal area near Qingdao bathing beach. Iron chloride hexahydrate ($FeCl_{3}$·6$H_{2}$O, 99%), tannic acid, triethylamine (99%) and octadecylamine (90%) were purchased from Shanghai Macklin Biochemical Co., Ltd. (Shanghai, China) Sodium hydroxide and ethanol were purchased from Shanghai Wokai Biotechnology Co., Ltd. (Shanghai, China).

### 2.2. Synthesis of Modification of EP

Pre-treatment of EP: To desalinate and remove impurities, the crude EP fibers were immersed in fresh water for 30 min, and then washed with distilled water. This soaking and washing process was repeated three times, after which the EP fibers were dried at 80 °C overnight for further use. Preparation of modified EP: 400 mg Enteromorpha fibers were placed in a 100 mL beaker, and 10 mL Fe$Cl_{3}$·6$H_{2}$O aqueous solution was added. After stirring for 1 min, 40 mL of tannic acid solution was added, and the mixture was stirred at room temperature for 5 min. The concentration ratio of Fe$Cl_{3}$·6$H_{2}$O solution to tannic acid solution was optimized. Adjusting the pH to 8 by using 0.1 mol L^−1^ NaOH solution and stirring continually at room temperature for additional hour. Subsequently, the EP fibers were collected by removing the solvent and washed extensively with deionized water and ethanol, and then air dried. The obtained Enteromorpha fibers were then placed in an ethanol aqueous solution containing octadecylamine (170 mg octadecylamine, 6 mL deionized water, 14 mL ethanol), and the pH was adjusted to 8.5 with triethylamine. The mixture was stirred at 30 °C for 2 h. Finally, highly hydrophobic Enteromorpha fibers were obtained by removing the solvent, washing multiple times with ethanol, and baking in an oven at 80 °C for 5 h.

### 2.3. Oil Adsorption Experiments

A specific volume of peanut oil was added to a beaker filled with water to simulate an oil spill on the sea surface. A dry blank stainless-steel basket was prepared. A total of 1 g of dry unmodified and modified Enteromorpha fibers was weighted and placed in the stainless-steel basket. The basket was the immersed in the beaker containing the floating oil. To ensure full contact and absorption of Enteromorpha fibers with oil, the mixture was stirred with a glass rod and allowed to soak for 5 min. Afterward, the basket was lifted and allowed to stand for 30 min to remove any residual oil and water on Enteromorpha fibers. The weight of the basket was recorded as *M*2. The treated basket underwent the same procedure, and its weight recorded as *M*1. The oil absorption performance of unmodified and modified EP was calculated using the following equation:

(1) M3=(M2−M1)/1

Here, *M*3 (g $g^{-1}$) represents the adsorption capacity of Enteromorpha fibers to oil.

### 2.4. Oil–Water Mixture or Emulsion Separation

Preparation of oil–water emulsions: 200 mL of peanut oil and 10 mL of distilled water were added to a 250 mL beaker, along with 0.21 g of sodium dodecyl sulfate as a stabilize. The mixed solution was vigorously stirred for 3 h to obtain a dispersed and uniform emulsion, which was then stored for later use. Notably, there was no obvious stratification observed in the prepared oil–water emulsion after one week of storage.

Separation of oil–water emulsions: In the simulated oil–water separation process, Enteromorpha fibers were fixed in a funnel device as a filter for separating the oil–water emulsion. Before separation, both unmodified and modified Enteromorpha fibers were pre-moistened with the corresponding oil and organic solvent, after which the prepared oil–water emulsion was poured into the funnel. The droplet state of the emulsion before and after separation was observed using an optical microscope.

### 2.5. Characterization and Measurement

The hydrophobicity and lipophilicity of the material before and after modification were investigated using contact angle measurement (CA, KRUSS, Hamburg, Germany). Each sample was tested in three different areas, and the results were averaged. The functional groups of the materials before and after modification were analyzed using Fourier-transfer-infrared spectroscopy (FTIR, ThermoFisher scientific, Waltham, MA, USA). FTIR measurements were conducted on a Nicolet iS10 spectrometer, scanning wavenumber ranging from 4000 to 400 cm^−1^. The specific surface area and pore size distribution were studied by using Micromeritics ASAP 2460 Specific surface area and pore size analyzer (ASAP, Micromeritics, Shanghai, China). The surface morphology of the materials before and after modification was examined using a field emission scanning electron microscope (SEM, Zeiss, Oberkochen, Germany). Digital photographs of the fibers were captured with a digital camera.

## 3. Results and Discussion

**Figure 1:**
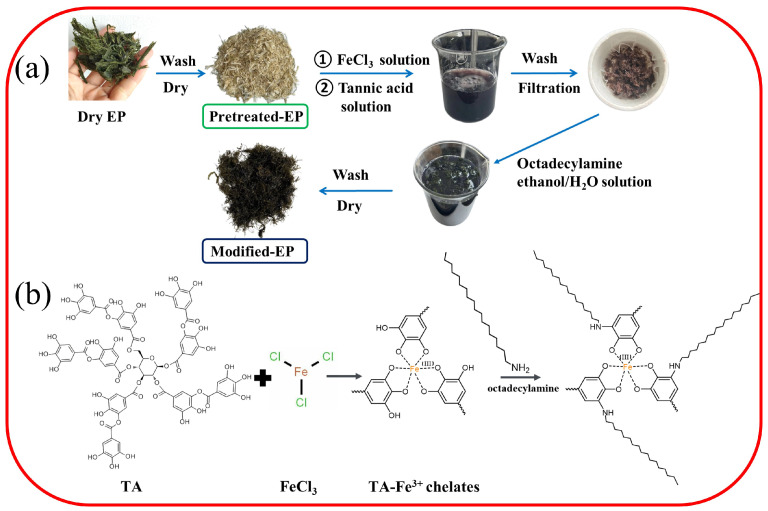
A schematic illustration of the (**a**) preparation procedure and (**b**) chemical reaction of TA-modified EP.

The modified EP fibers were fabricated by sequentially treating with Fe$Cl_{3}$-TA and ODA solution, and the synthesis process and possible chemical reactions as illustrated in Figure 1. Firstly, phenolic hydroxyl groups in tannic acid molecules react with iron ions to form network-structured complexes, which cover the surface of EP fibers. Subsequently, ODA is grafted onto the pre-treated EP fibers via an addition reaction between the residual catechol group in the TA-Fe$Cl_{3}$ complex and the amino group in ODA (Figure 1b), resulting in the desired high hydrophobicity and superoleophilic.

**Figure 2:**
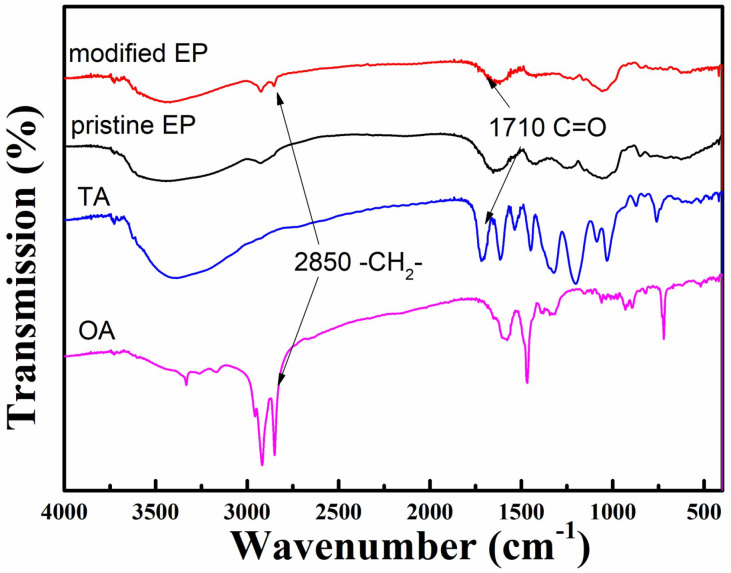
FTIR spectrum of pristine, modified EP, TA and ODA, respectively.

Moreover, the surface chemical states of both pristine and TA-modified EP fibers were analyzed using FTIR, as shown in Figure 2. The primary elements in EP fibers are carbon and oxygen, which form polysaccharide, carbohydrate, protein, contributing to their inherent hydrophilicity. In both pristine and treated EP fibers, peaks near 2984 and 3389 cm^−1^, corresponding to the -C$H_{2}$- and hydroxyl group on the polysaccharide chain, are observed. However, in the TA-modified EP, Compared to the pristine EP, the increased density of the peak around 1710 cm^−1^ band is attributed to the stretching vibration of -C=O in TA [23,24]. Additionally, an extra peak at 2850 cm^−1^ appears in the modified EP fibers, representing the -C$H_{2}$- vibration peak of octadecylamine, confirming the successful grafting of ODA onto the EP fibers.

**Figure 3:**
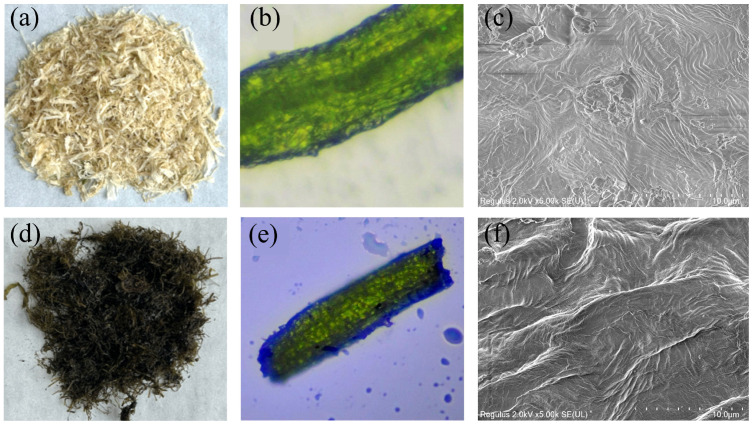
Photograph of (**a**) pristine and (**d**) modified EP. Optical microscopy of (**b**) pristine and (**e**) modified EP. SEM images of (**c**) pristine and (**f**) modified EP.

The fine structure and morphology of the unmodified and modified UP fibers were characterized using optical microscopy and scanning electron microscopy (SEM), as shown in Figure 3. From the optical image (Figure 3a,b), the overall micromorphology of EP fibers shows no significant change before and after treatment, apart from the color difference. The pristine UP fibers appear light yellow, while modified-EP fibers display a purple-black color due to the formation of a purple–black complex from the reaction between TA and hexahydrate Fe$Cl_{3}$, which coats the EP fibers surface. According to the optical microscopy images, both samples exhibit a green tubular structure with numerous air bubbles (Figure 3b,e), contributing to their ability to automatically float on the sea surface. SEM images (Figure 3c,f) provide a closer look at the surface morphology before and after modification. While the pristine UP fibers exhibit continuous and homogeneous sunken sites, the modified EP fibers are covered with irregular, rough impurities. These deposits indicate the successful coating of the tannic acid–iron complexes on the algae surface, which increases the surface roughness. This enhanced roughness is beneficial for raising surface energy, ultimately improving the fibers hydrophobic properties. It should be noted that the algae intrinsically have low surface area and pore volume, which cannot be regarded as porous materials [14]. However, the modified EP in our work displayed outstanding oil absorption, which thanks to the fibrous morphology and the voids in those fibrous bundles.

**Figure 4:**
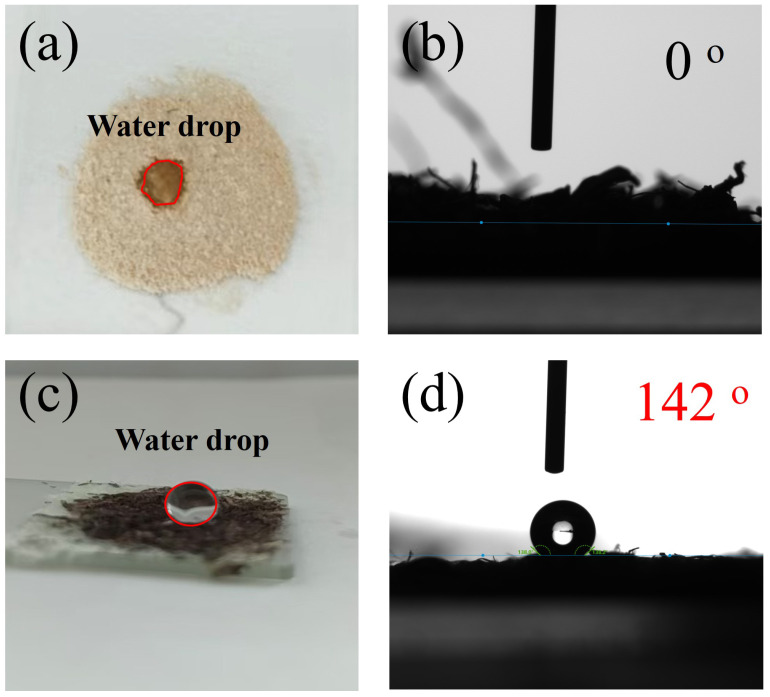
The wettability of pristine and the modified EP. image of water drop on pristine (**a**) and modified EP (**c**). Water contact angle for pristine (**b**) and modified EP (**d**).

The water contact angle (WCA) is a crucial indicator for assessing the surface wettability of materials. Therefore, the changes in wettability of EP fibers before and after TA modification were investigated using WCA measurements. As shown in Figure 4, when a water droplet contacts the surface of pristine EP fibers, it is quickly and completely absorbed into the matrix, resulting in a contact angle close to 0°, demonstrating superhydrophilicity. After modification, however, a dramatic change is observed, with the wettability shifting from superhydrophilicity to hdyropphobic (Figure 4c,d). The modified EP fibers exhibit a contact angle as large as 142°, indicating their low affinity for water. Notably, the air trapped within the fiber bundle likely contributes to their hydrophobic behavior [10]. Combing the synergistic effect from the trapped air in bundle with the hydrophobic ODA groups on the surface, the as-modified EP achieve ultra-hydrophobicity.

**Figure 5:**
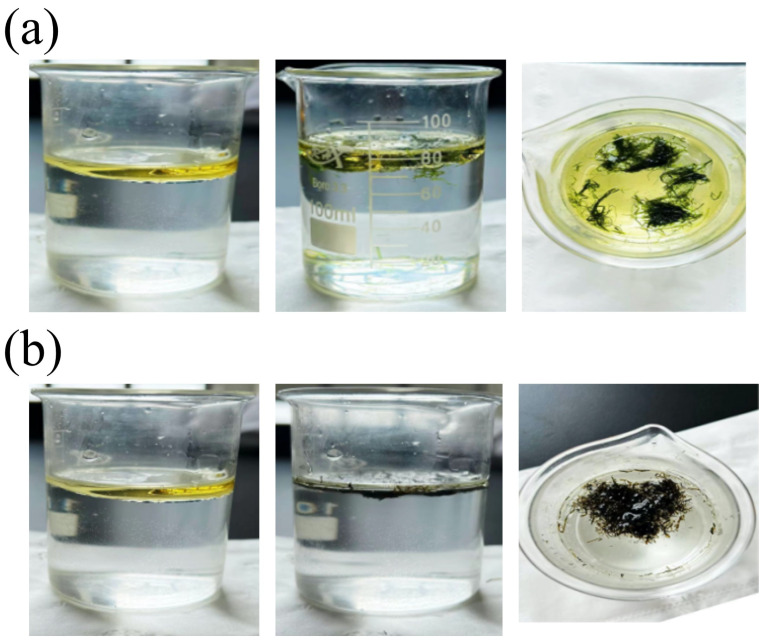
The absorption of the (**a**) pristine EP and (**b**) modified EP to oil from seawater.

Given that the material is intended for oil–water separation, it is essential to evaluate its selective towards oil and water. The oil–water selectivity of the pristine and modified EP was assessed by observing their ability to absorb peanut oil from seawater in a beaker (Figure 5). As shown in Figure 5a, the pristine EP fibers remained dispersed in the oil layer when placed in the oil–water mixture, showing no selective absorption between water and oil due to their inherent superhydorphilic and sueroleophilic nature. As a result, the pristine EP fibers cannot selectively absorb oil from water, making them unsuitable for oil–water separation. In contrast, when the modified EP were placed in seawater, they floated on the surface with only minimal water immersion, indicating their low water affinity. Simultaneously, the fibers adsorbed all oil floated on the surface and aggregated, demonstrating excellent oil absorption and hydrophobic properties (Figure 5b).

For an effective oil-absorption material, it must exhibit both superoleophilic and ultrahydrophobic to prevent simultaneous absorption of oil and water. To further characterize the absorption properties of the pristine and modified EP fibers, their uptake in oil–water mixtures, pure water, and pure oil was measured and summarized in Table 1, with 1 g EP fibers used in each experiment. The results indicate that the pristine EP fibers exhibited high water absorption (7.9 g $g^{-1}$), and relatively poor oil absorption (23.6 g $g^{-1}$). The high water affinity of the pristine fibers is due to the abundance of hydroxyl groups on their surface, leading to greater water absorption than oil. In contrast, the modified EP fibers displayed high oleophilicity and good hydrophobicity, with oil absorption reaching 63.4 g $g^{-1}$ and water absorption reduced to 1.4 g $g^{-1}$. The oil absorption of the modified EP fibers in oil–water mixtures increased by 62.8%, while hydrophobicity improved by 82%, compared to the untreated samples. These findings demonstrate that the modified EP fibers possess excellent oil absorption capabilities and superior hydrophobicity, making them highly effective for collecting oil from seawater.

**Figure 6:**
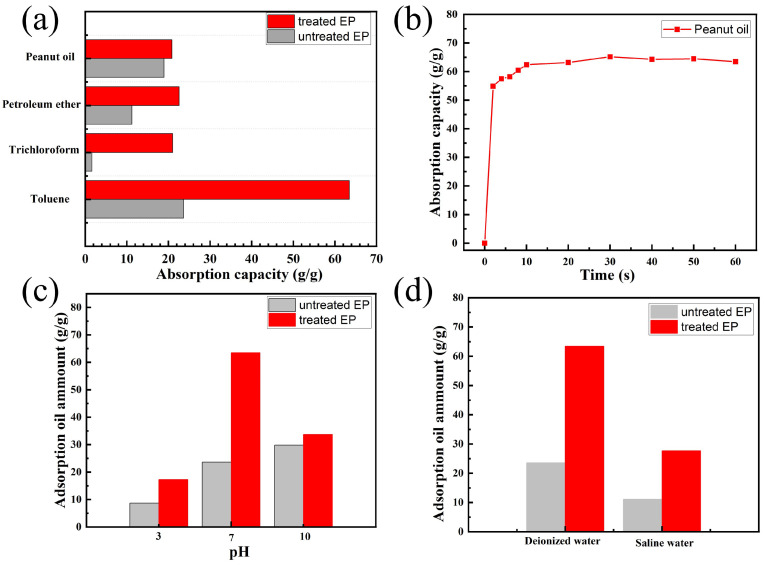
(**a**) The absorption capacity of the pristine and modified EP for peanut oil and different organic solvents. (**b**) Adsorption rate of modified EP for peanut oil. The adsorption capacity of the pristine and modified EP at (**c**) different pH and (**d**) saturated saline water.

The peanut oils and several organic solvents were selected to the absorption capacity experiment as follows: peanut oil, petroleum ether, chloroform, and toluene. The adsorption value of treated EP to various oils and organic solvents is range of 20–64 g $g^{-1}$ (Figure 6a), which is much larger than that of untreated EP (2–24 g $g^{-1}$). The superior adsorption capacity of treated EP for various oils and organic solvents is attributed to their high porosity and surface lipophilicity. Furthermore, to investigate the adsorption kinetic of treated EP, the change in adsorption capacity over time was plotted as shown in Figure 6b. For high viscosity peanut oil, the saturation adsorption time is about 10 s and most oils were absorbed within the first 2 s, which is consistent with other reports [19].

Since the pH value and ionic strength is one important factor for the oil adsorption behavior, we studied the adsorption capacity of treated and untreated EP for different pH values (pH = 3.7, 7, and 10.2) and saturated saline water. As shown in Figure 6c, the adsorption capacity of treated EP for peanut oils was 17.3 g $g^{-1}$ (pH = 3.7), 63.4 g $g^{-1}$ (pH = 7), and 33.7 g $g^{-1}$ (pH = 10.2), respectively. High and low pH value are both not beneficial to oil adsorption. In high ionic solution, the adsorption capacity of treated EP is about 27.7 g $g^{-1}$, which is lower than that of di-ionized water (Figure 6d).

**Figure 7:**
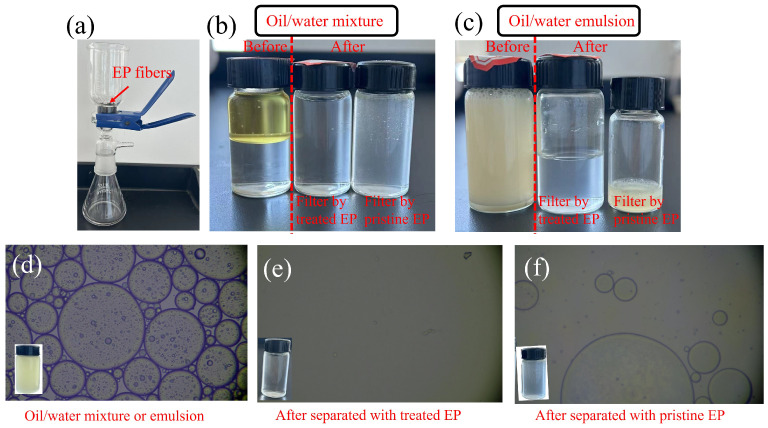
(**a**) The image of filtration device. The image of oil/water mixture (**b**) and oil/water emulsion (**c**) in the bottle before and after filtration. The optical microscopy images of oil–water emulsion before (**d**) and after filtration with modified EP (**e**) and pristine EP (**f**).

Moreover, the modified EP demonstrated efficient oil–water separation capabilities due to their remarkable ability to differentiate between oil and water. As illustrated in Figure 6, the modified EP fibers were used as a filter cartridge in a sand core filtration device to separate oil–water mixtures (Figure 7a). The mixture was poured from above and allow to flow naturally into a glass beaker below through the EP fibers, without the need for any external force. Due to the strong hydrophobicity of the modified EP fibers, water was repelled and fell into the beaker below under gravity, while the oil was adsorbed and retained by the EP fibers above the sand core. By comparing the collected liquid before and after filtration (Figure 7b), it was evident that the oil and water were successfully separated by the pristine and modified EP filter cartridge. Differently, the liquid filtered through pristine EP fibers appeared more turbid (Figure 7b the bottle on the right), showing visible oil droplets at the macroscopic level.

Since spilled oil on the sea surface typically forms an oil–water emulsion instead of an oil–water mixture, due to wind and wave actions, oil–water emulsion separation experiments were also conducted. As shown in Figure 7c, light yellow, creamy oil–water emulsions became a relatively clear liquid after filtration through the modified EP fibers, demonstrating their excellent separation performance for oil-in-water emulsions. Notably, when untreated EP fibers were used as the filter, the emulsion remained above the sand core and could not be filtered.

Additionally, the liquid before and after separation were also observed under an optical microscope, as shown in Figure 7d–f. Figure 7d reveals densely distributed oil droplets of varying sizes in the unseparated oil–water mixture or emulsion. After filtration, the number of oil droplets in the obtained liquid decreased significantly (Figure 7e,f), indicating that EP fibers have a certain oil adsorption capacity, mainly due to their hollow fiber structure. However, obvious oil droplet distribution was still visible in the collected liquid filtered through pristine EP fibers, reflecting their poor oil–water separation performance (Figure 7f). In contrast, the liquid obtained from the filtration with TA-modified EP fibers contained almost no visible oil droplets under the microscope, confirming their excellent separation efficiency (Figure 7f).

From a practical perspective, considerations such as the cost, availability of resources, and environment compatibility of the raw materials are critical for oil collection. Several artificial and natural materials, such as sponges, kapok fibers, and cotton, were selected as the representatives for comparison with the EP fibers harvested from green tides. Sponges, as artificial oil absorbers, are notable for their high absorption capacity, reaching 80–130 g $g^{-1}$ for common oil [25,26]. However, their high cost (USD 250 per cubic meter) limits their practical usage for large-scale oil collection. Natural oil sorbents, like kapok fibers and cotton, also have high costs, ranging from USD 2000 to 6000 per ton. Additionally, their limited availability poses another challenge for their widespread application in oil spill treatment. In comparison, the EP fibers derived from green tides outbreaks offer significant advantages in terms of both availability and cost. Since the modified EP in this work are fully bio-based raw materials, with excellent biodegradability and renewability, the used treated EP can be buried in soil for natural degradation.Therefore, due to their biocompatibility and biodegradability, EP fibers provide benefits not only to the environment but also to the local economy and ecology. Notably, in this work, TA, iron (iii) chloride (FeCl_3_) and OA were chosen as the reactive monomers and dissolved in green solvent aqueous phase or ethanol, which are all non-toxic materials or regents and can be qualified as environmentally friendly.

## 4. Conclusions

The potential for using natural materials for spill oil sorption and recovery is extensive. Enteromorphere prolifera, a type of algae, causes significant environmental disruption during green tide outbreaks. Therefore, exploring new applications of EP fibers, and transforming this natural disaster into a valuable resource for humanity, is highly desirable. In this study, we modified EP fibers with TA-Fe$Cl_{3}$ and ODA, resulting in superoleophilic and hydrophobic material suitable for oil sorption. The modified EP fibers exhibited an oil absorption capacity of approximately 63.4 g $g^{-1}$ and a water absorption capacity of 1.4 g $g^{-1}$, representing an 62.8%, increase in oil absorption and an 82%, increase in hydrophobicity compared to untreated EP fibers. In summary, utilizing these highly hydrophobic and oleophilic EP fibers for oil spill collection effectively repurposes green tide algae. This approach not only addresses the issue of algae reuse, and reduces reliance on organic synthetic oil-absorbing materials, but also offers a cost-effective solution due to their abundant resources and straightforward modification process. Importantly, the modified EP fibers are low in toxicity and easy to biodegrade, posing no risk of secondary pollution to the environment. We believe that this study will be helpful to broaden the application of natural biomass materials in oil spill treatment.

## Figures and Tables

**Table 1:** The absorption of pristine and modified EP on oil–water mixture, pure oil, and pure water, respectively.

Material	Oil–Water Mixture (g/g)	Pure Oil (g/g)	Pure Water (g/g)
Pristine EP	12.6	23.6	7.9
Modified EP	9.7	63.4	1.4

## Data Availability

The original contributions presented in the study are included in the article, further inquiries can be directed to the corresponding authors.
